# Invasive Pneumococcal Pneumonia and Respiratory Virus Co-infections

**DOI:** 10.3201/eid1802.102025

**Published:** 2012-02

**Authors:** Hong Zhou, Michael Haber, Susan Ray, Monica M. Farley, Catherine A. Panozzo, Keith P. Klugman

**Affiliations:** Emory University, Atlanta, Georgia, USA (H. Zhou, M. Haber, S. Ray, M.M. Farley, K.P. Klugman);; University of North Carolina, Chapel Hill, North Carolina, USA (C.A. Panozzo)

**Keywords:** Pneumococcal diseases, pneumonia, influenza, H3N2, respiratory syncytial virus, temporal association, viruses

## Abstract

To confirm whether respiratory virus infections increase susceptibility to invasive pneumococcal pneumonia, we examined data from 11 influenza seasons (1994–2005) in the United States**.** Invasive pneumococcal pneumonia was significantly associated with influenza and respiratory syncytial virus activities in 5 seasons. Association strength was higher when strain H3N2 was the predominant influenza A virus strain**.**

## Abstract 2

Each year, especially in the winter, many get sick and some die of invasive pneumococcal pneumonia. Does this type of pneumonia increase in the winter because people are in closer contact indoors?  Or are people more susceptible to this bacterial disease after having had a seasonal respiratory virus infection?  A season-by-season analysis found an association between pneumococcal pneumonia and two viruses (influenza and respiratory syncytial virus). The association varied by season and was strongest when the predominant influenza virus subtype was H3N2. Vaccination against influenza and RSV should also help protect against pneumococcal pneumonia.

Invasive pneumococcal pneumonia (IPP) diseases cause high rates of illness and death every year. Existing evidence supports the biological plausibility that preceding respiratory viral infections, particularly with influenza virus and respiratory syncytial virus (RSV), increase susceptibility to IPP diseases (*1*). Although it has been generally believed that the increases in IPP diseases in winter relate to increased activity of respiratory viruses, especially influenza virus and RSV (*2**–**6*), evidence of association of IPP diseases and respiratory virus infections is not conclusive. If such an association is likely, then public health authorities should emphasize that vaccination against influenza, as well as other interventions against influenza and RSV, can reduce incidence of IPP diseases. We believe that cold temperatures, lack of sunshine, and rainy and snowy weather are the main reasons that persons increase their indoor activities during the winter and that respiratory diseases are mainly transmitted by close person-to-person contact (*5**,**7**,**8*). Therefore, we conducted separate analyses of the association of IPP with seasonal influenza virus and RSV activities, adjusted by climate variables, for each of 11 influenza seasons from 1994–95 through 2004–05.

## The Study

All data used in this study were recorded for each influenza season (surveillance weeks 40–20) from 1994–95 through 2004–05. Weekly IPP cases were obtained from the Active Bacterial Core Surveillance for Georgia Health District 3. Cases of IPP were defined by isolation of *Streptococcus pneumoniae* from normally sterile sites, e.g., cerebrospinal fluid or blood. Only IPP case-patients (defined as persons with *S. pneumoniae* isolated from pleural fluid or persons with a clinical diagnosis of pneumonia and *S. pneumoniae* isolated from blood or another sterile body site*)* were included in this study. Influenza virus surveillance data were obtained from the World Health Organization. Weekly data were collected from each state from October through May of each influenza season. The influenza virus–positive isolate percent was defined as the percentage of influenza virus–positive isolates out of all influenza specimens. Percentages of isolates positive for influenza A (H1N1), A (H3N2), and B viruses were also calculated.

Weekly RSV data were obtained from the National Respiratory and Enteric Virus Surveillance System (www.cdc.gov/surveillance/nrevss/). For each season, hospitals and laboratories reported to the Centers for Disease Control and Prevention the numbers of specimens tested for RSV by antigen detection each week. The RSV detection percent was defined as the percentage of RSV-positive isolates out of all RSV specimens. The influenza virus and RSV data used in this study were from the US Census South Atlantic Region (www.census.gov/geo/www/us_regdiv.pdf). Data on daily mean temperature (in ^o^F), total sunshine (in hours), and total precipitation (in inches) were obtained from the National Weather Service Atlanta regional weather center at Hartsfield-Jackson International Airport (*9*). The weekly mean temperature, total sunshine, and total precipitation were calculated from daily data.

For each of the 11 influenza seasons, we applied negative binomial regression models with multiple predictors to relate the weekly IPP rates with the indicators of influenza and RSV activities while adjusting for the weekly mean temperature, total sunshine, and total precipitation. The full model used to explore the association of IPP with influenza and RSV was as follows:

log(*Y_t_*) = α + β_0_*X_t_* + β_1_*X_t_*_−1_ + β_2_*X_t_*_−2_ + γ_0_*Z_t_* + γ_1_*Z_t_*_−1_ + γ_2_*Z_t_*_−2_ + λ*W_t_* + η*U_t_* + θ*V_t_* + log(*N_t_*)

where for week *t*, *Y_t_* is the incidence of IPP, *X_t_* is the influenza virus–positive isolate percent, *Z_t_* is the RSV detections percent, *W_t_* is the mean temperature, *U_t_* is the total sunshine, *V_t_* is the total precipitation, and *N_t_* is the population size. The reduced model excluded the influenza and RSV terms. We calculated the difference in the log-likelihood ratio statistic between the full and reduced models and used it to test the hypothesis that IPP incidence is not associated with influenza and RSV activities when weekly temperature, sunshine, and precipitation were adjusted for.

For influenza seasons 1994–95 through 2004–05, the average annual incidence of IPP was 19.78 per 10 million persons (Table 1). Table 1 also shows the means of total number of IPP cases; influenza-positive isolates; RSV detections; and the average temperature, sunshine, and precipitation for each of these influenza seasons.

**Table 1:** IPP incidence and associated factors, 11 seasons, Atlanta, Georgia, USA*

Season	No. IPP cases	No. IPP cases/10 million population	No. influenza isolates	No. influenza isolates/100 specimens	No. RSV detections	No. RSV detections/100 specimens	Temperature, ^o^F†	Precipitation, inches‡	Sunshine, h‡
1994–95	633	26.75	1,203	12.67	2,116	23.41	57.44	0.81	79.46
1995–96	643	26.54	916	11.76	2,016	26.25	52.31	1.31	79.38
1996–97	653	26.33	1,382	15.87	1,635	18.64	55.24	0.89	79.30
1997–98	673	25.73	1,876	19.46	1,682	16.83	53.09	1.16	79.78
1998–99	599	23.04	2,073	21.01	1,672	15.61	56.42	0.59	79.60
1999–00	600	22.57	1,532	16.29	878	22.31	55.53	0.62	79.51
2000–01	516	18.97	1,409	15.31	3,458	21.45	52.97	0.85	79.43
2001–02	342	12.34	2,045	20.44	2,001	15.70	56.08	0.66	79.34
2002–03	336	11.90	1,287	14.81	1,713	14.23	53.85	1.18	79.27
2003–04	360	12.19	4122	28.69	4,227	18.50	55.07	0.63	79.75
2004–05	328	11.25	3813	23.34	3,415	14.34	54.94	1.08	79.56
Total	5683	NA	21,658	NA	24,813	NA	NA	9.78	874.37
Mean	517	19.78	1,969	NA	2,256	NA	54.81	0.89	79.49

*Virus data from US Census South Atlantic Region (www.census.gov/geo/www/us_regdiv.pdf). IPP, invasive pneumococcal pneumonia; RSV, respiratory syncytial virus; NA, not applicable. †Seasonal mean calculated from weekly means. ‡Seasonal mean calculated from weekly totals.

Table 2 shows the result of the negative binomial regression analysis for each influenza season. Significant associations between IPP and both influenza-positive isolate percent and RSV detection percent were found for 5 of the 11 seasons. Table 2 also shows the percentages of the influenza A (H1N1), A (H3N2), and B virus strains in each season. The predominant influenza virus strain (percentage >50%, [*10*]) varied across seasons. Influenza A (H3N2) virus was predominant in 8 seasons, and influenza B virus was predominant in 2 seasons. One season had no predominant influenza virus strain. Table 2 also shows a significant association between IPP incidence and influenza virus and RSV activities in 5 of the 8 seasons in which A (H3N2) was the predominant influenza virus strain; there was no significant association in any of the 3 seasons in which influenza A (H3N2) virus was not predominant (p = 0.12, 2-sided mid–p-value exact test).

**Table 2:** Negative binomial regression analysis of the association of invasive pneumococcal pneumonia weekly incidence with influenza and respiratory syncytial virus activities *

Season	p value†	% Influenza A (H1N1)	% Influenza A (H3N2)	% Influenza B
1994–95	**0.029**	1.05	58.98‡	39.96
1995–96	0.2	36.58	30.39	33.03
1996–97	**0.002**	0.58	50.29‡	49.12
1997–98	0.09	0.19	98.66‡	1.15
1998–99	**0.015**	1.04	58.40‡	40.55
1999–00	0.424	3.8	95.75‡	0.45
2000–01	0.069	46.88	1.04	52.08‡
2001–02	0.641	12.09	74.53‡	13.38
2002–03	0.649	30.22	4.64	65.14‡
2003–04	**0.004**	0.03	98.75‡	1.22
2004–05	**<0.001**	0.19	69.97‡	29.85

*Analysis adjusted by temperature, sunshine, and precipitation. p values based on the likelihood ratio test for lack of association. †**Boldface** indicates a statistically significant association. ‡Predominant (>50%) influenza strain.

To demonstrate the association of IPP incidence with influenza virus and RSV activities and the climate data, we displayed the weekly IPP, influenza virus, RSV, temperature, sunshine, and precipitation data for seasons 1998–99 and 2003/04 (Figure). Similar figures for the other 9 seasons can be found in the Technical Appendix.

**Figure:**
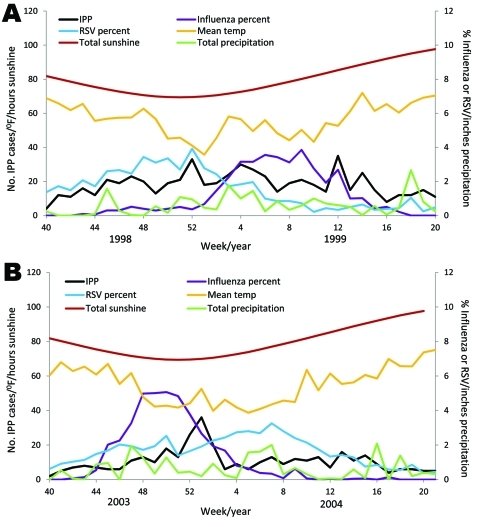
Trends for invasive pneumococcal pneumonia, virus, and climate data for 1998–99 (A) and 2003–04 (B), United States. IPP, invasive pneumococcal disease; influenza percent, percentage of influenza virus–positive isolates out of all influenza specimens; RSV, respiratory syncytial virus; RSV percent, percentage of RSV-positive isolates out of all RSV specimens.

## Conclusions

To explore the temporal variability in the association of IPP incidence with influenza virus and RSV activities over a long period, we conducted a season-by-season analysis over 11 influenza seasons. We adjusted the association of IPP with influenza virus and RSV by temperature, precipitation, and sunshine because these factors are believed to be associated with pneumococcal and viral respiratory infections (*5**,**7**,**8*).

The results indicated substantial variability across seasons in the strength of association of IPP incidence with influenza virus and RSV activities. This variability explains why findings from previous studies (*2**–**5**,**11*), which were based on data from a single season or on combined data from several seasons, are inconsistent. We found significant associations of IPP incidence with influenza virus and RSV activities for 5 of 11 influenza seasons from 1994–95 through 2004–05. Notably, in each of the 5 seasons for which we found a significant association, influenza A virus strain H3N2 was predominant (this strain predominated in 8 seasons). Alternatively, we did not find a significant association in any of the 3 seasons in which strain A (H3N2) was not the predominant influenza virus strain. Although this difference was not statistically significant, it suggests that the association between IPP and influenza virus and RSV activities might be stronger in seasons in which strain A (H3N2) is predominant. This finding is consistent with the idea that excess neuraminidase expression of strain H3N2 compared with strain H1N1 influenza viruses may lead to an excess number of pneumococcal superinfections (*12*).

In summary, in this season-by-season analysis, we found substantial variation in the strength of the association of IPP incidence with influenza virus and RSV activities across seasons. We also found that this association may be associated with the predominant influenza virus strain. More studies, using data from more seasons and from several geographic areas, are needed to better explain the variation in the association between invasive pneumococcal diseases and respiratory viral infections.

## Supplementary Material

Technical Appendix Trends for invasive pneumococcal pneumonia, viraus, and climate data during 9 influenza seasons, United States.
